# Oswald Avery: Pioneer of Bacterial Vaccines and the First to Discover the Function of DNA

**DOI:** 10.7759/cureus.71465

**Published:** 2024-10-14

**Authors:** Ger Rijkers, Karen Dekker, Guy Berbers

**Affiliations:** 1 Science Department, University College Roosevelt, Middelburg, NLD; 2 Centre for Infectious Disease Control, National Institute for Public Health and the Environment, Bilthoven, NLD

**Keywords:** dna, polysaccharide conjugate vaccine, streptococcus pneumoniae, transformation, urine test

## Abstract

Dr. Oswald Theodore Avery (1877-1955), M.D., played an immense role in the biological and medical field because of his groundbreaking research. All his life, he worked on *Streptococcus* *pneumoniae*, the bacterium that causes pneumonia. He discovered the principle of polysaccharide conjugate vaccines, which now save the lives of millions of children and the elderly. He found that bacteria can transfer plasmids; most importantly, he identified that deoxyribonucleic acid (DNA) is the carrier of genetic information instead of the (at that time widely accepted) protein theory. Though he was never awarded the Nobel Prize, his work was the basis for countless achievements building on his foundation, ranging from effective vaccines to gene transfer technologies.

## Introduction and background

**Figure 1:**
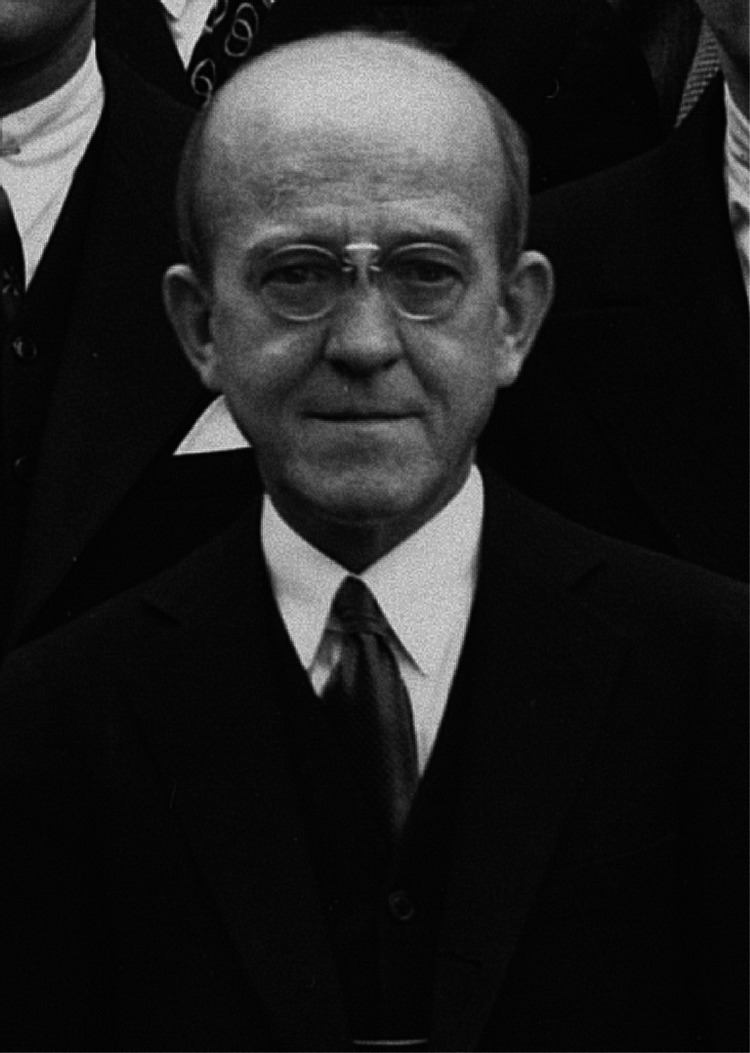
Dr. Oswald T. Avery (1877-1955) Illustration cropped from a Rockefeller Institute for Medical Research staff photograph, 1937 Source: Wikimedia Commons File: Oswald T. Avery Portrait 1937 [1] Author: Unknown author Permission: Public domain This image is a work of the National Institutes of Health, part of the United States Department of Health and Human Services, taken or made as part of an employee's official duties. As a work of the U.S. Federal Government, the image is in the public domain. Accessed September 2, 2024.

The main purpose of this historical vignette is to credit Dr. Oswald T. Avery (Figure 1) for his immense contributions to the medical, microbiological and immunological world [1]. Avery was an immunologist and bacteriologist, working at the Rockefeller Institute from 1913 to 1948, who published compelling evidence that deoxyribonucleic acid (DNA), a molecule with an unknown function at that time, was the carrier of genetic information within the cell. Previously, the consensus was that proteins were the location of genes and heredity. DNA, composed of just four different building blocks, was believed to be incapable of encoding all genetic information. Proteins, composed of 20 different amino acids, would be the ideal molecules to harbour all genes. Avery discovered that the “transforming substance” responsible for the pneumococcal bacteria’s ability to change its genetic expression was not protein at all, but DNA [2]. This principle, based on his original work, is now used daily, to genetically modify bacteria, in order to produce proteins for human use. Although he accomplished a great many achievements in the realm of medicine and received many accolades, he was not awarded the Nobel Prize.

## Review

Life and career

Oswald Avery was born to Elizabeth Crowdy and Joseph Francis Avery on October 21, 1877, in Halifax, Canada. The second of three, he was preceded by his brother Ernest and succeeded by his brother Roy. In 1887, at 10 years old, the Avery family moved to New York City (NYC), USA. His father was a Baptist pastor at the Mariners Temple Baptist Mission Church in NYC, but disaster struck the family in 1892 when Ernest passed away at 18 from suspected tuberculosis. Some months later, Joseph Avery passed away too, leaving Oswald Avery to raise his younger brother Roy with his mother [3].

Oswald Avery began his education at the New York Male Grammar School, followed by a Bachelor’s degree in music and the humanities at Colgate in 1900, where he also was the leader of the University Band. He followed his academic career degree by enrolling in the College of Physicians and Surgeons in New York, where he received his medical degree in 1904. Because he felt that as a clinician he could do little for his patients in terms of treatment, he wanted to research the mechanisms of disease, especially infectious diseases.

His first appointment as a researcher was at the Hoagland Laboratory in Brooklyn NYC in 1907, where he became associate director of the division of bacteriology. He worked together with Benjamin White until White developed tuberculosis himself. Avery then started research on tuberculosis, resulting in several publications [4,5] that caught the attention of Rufus Cole, the director of the Hospital of the Rockefeller Institute for Medical Research. He offered Avery a job, which he accepted in 1913.

The focus of the research in the hospital was to develop antiserum to be used as a passive vaccination and treatment of lobar pneumonia. At that time, pneumonia killed over 50,000 persons, mainly the elderly, every year in the U.S. The most common cause of lobar pneumonia is an infection of the lungs with *Streptococcus pneumoniae*, spread by close contact, coughing or sneezing. The development of an “all-inclusive” pneumococcal antiserum was complicated by the recognition by Fred Neufeld at the Robert Koch Institute in Berlin of distinct types of pneumococcus, reviewed in the study by Eichmann and Krause [6], which would require type-specific antibodies.

When the United States entered the First World War in 1917, the research activities of Avery on pneumococcal infections were put on hold when he joined the U.S. Army Medical Corps. Because he was born in Canada and formally a British subject, he was designated a private. Because of his service in the Medical Corps, Avery qualified for U.S. citizenship in 1918 and was promoted to the rank of captain. During the war, he did research on the emerging influenza pandemic. When the war ended, Avery immediately returned to his laboratory at Rockefeller to continue with his studies on pneumococci.

Avery spent 35 years at the Institute, sharing an apartment with Alphonse R. Dochez as they worked together to understand the bacterium *Streptococcus pneumoniae* (pneumococcus), at that time named *Diplococcus pneumoniae* [7].

Avery was forced to officially retire at the age of 65 in 1943, but he continued his research at the Institute until 1948. He then moved to Nashville, Tennessee, to be closer to his brother Roy and Roy’s family. Roy had followed in Oswald’s footsteps as a bacteriologist at the Vanderbilt School of Medicine. On February 20, 1955, Oswald Avery died at the age of 77 from liver cancer, surrounded by his family.

Discovery of DNA as the carrier of genetic information

Avery’s biggest achievement was his discovery that proteins were not the source of genetic material in the cell, but DNA. The discovery was made while working at the Rockefeller Institute, where he was appointed specifically to the pneumonia research program so that he could focus entirely on pneumococcus bacteria [8]. Avery’s crowning glory would happen in 1944, when he, along with Colin MacLeod and Maclyn McCarty, published a paper showing evidence that purified DNA was capable of the transformation of pneumococcal serotypes from a nonvirulent strain (rough, R) to a virulent one (smooth, S) [2]. The smooth appearance of a colony under the microscope is caused by the polysaccharide capsule of the bacteria. The capsule protects the bacteria from the immune system, and smooth pneumococci are therefore virulent. Rough pneumococci are unable to form capsular polysaccharides due to mutations in one or more of the enzymes involved in the synthesis of the polysaccharide. Rough pneumococci therefore are not virulent. Transformation of R to S can occur in vivo, which was demonstrated by Griffith in 1928 [9]. He injected mice with live R-form bacteria or with heat-killed S bacteria, and in both groups, all animals survived. Live S bacteria killed all mice. However, a mixture of live R bacteria with heat-killed S bacteria also killed most of the mice, and live S bacteria could be cultured from their blood [9]. Somehow, the transformation of R to S had occurred. Oswald Avery found it difficult to understand, even to believe these data and was of the opinion that a mistake was made. He therefore set out to repeat the Griffith experiments in vitro. The experiments performed by Avery, MacLeod, and McCarty showed that transformation could (also) be demonstrated in vitro, and more importantly, was based on DNA. They showed that transformation was aborted when their material was treated with enzymes that break down DNA, but neither protein-degrading enzymes nor RNA-degrading enzymes had any effect. All physical and chemical characteristics of the active product pointed to DNA. Every result led to the same conclusion: the transforming principle was based on the transfer and uptake of DNA [2].

**Figure 2:**
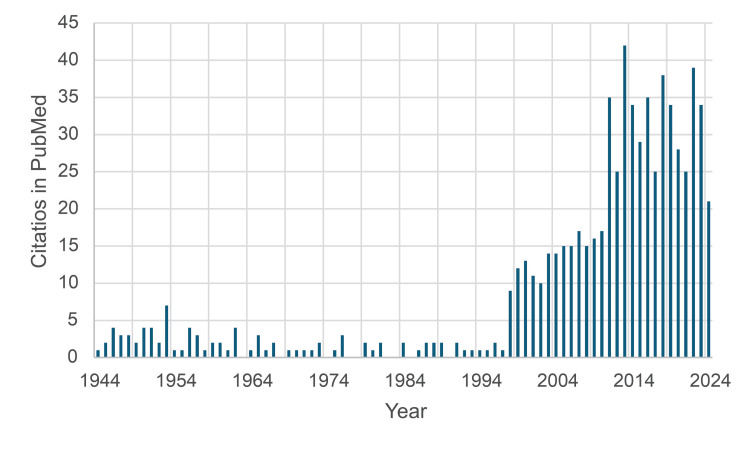
Citation score over time of Avery O, Macleod C, McCarty M: Studies on the chemical nature of the substance inducing transformation of pneumococcal types : induction of transformation by a desoxyribonucleic acid fraction isolated from pneumococcus type III Number of citations of Avery et al. [2] in PubMed

Although their discovery was clear to them, the paper was hesitant to declare it outright. Their conclusion says: The evidence presented supports the belief that a nucleic acid of the desoxyribose type is the fundamental unit of the transforming principle of pneumococcus Type III. The phrasing “supports the belief” is rather modest while they could have stated that the data unequivocally show that DNA and not protein carries genetic information. The paradigm at that time was that protein was the carrier of genetic information [10], but Avery was adamant in private that their discovery was groundbreaking [11]. In the first years after publication, the paper was hardly cited by others (Figure 2).

Avery indeed was correct, as the discovery paved the way for a deeper understanding of DNA, genes, and gene modification that would not have been possible based on the protein theory. Avery's paper [2], published in 1944, 80 years after the initial publication, has been cited 677 times. Remarkably, the vast majority of citing articles (93%) are from after 1994. It could be stated that Avery was 50 years ahead of time (Figure 2).

The principles discovered by Avery are widely used in modern biology and have many medical applications. As an example, we take the treatment of diabetes. Worldwide millions of people suffer from diabetes type 1 [12]. Their life depends on substitution with human insulin [13]. Thus, Avery’s work is crucial for the survival of these patients because his discovery of the “transforming principle” directly led to bacteria-synthesized insulin. Similar to the transformation of pneumococcal serotypes from R to S in his report, the DNA encoding for the human insulin gene is inserted into a bacterial plasmid and subsequently introduced into *Escherichia coli*. These transformed bacteria then produce human insulin, which is then harvested, purified, and used by diabetics to regulate their blood sugar [14]. The 1980 Nobel Prize in Chemistry was split between Paul Berg for his work on recombinant DNA technology and Wally Gilbert and Fred Sanger for DNA sequencing. Insulin thus was the first human therapeutic derived using recombinant DNA technology [15].

Polysaccharide conjugate vaccines

Avery’s other major discovery, predating his discovery that DNA is the carrier of genetic information, is no less important in saving the lives of (millions of) children, the elderly, and the immunocompromised.

In their initial experiments set out to develop specific antisera for the treatment of pneumococcal infections, Avery and Dochez immunized rabbits with heat-killed pneumococci. This induced antibodies, which when added in vitro to live bacteria slowed down the growth of pneumococci. They attributed this effect to an inhibition of bacterial enzymes and proposed the term antiblastic immunity [16]. Subsequent studies by others could confirm the growth inhibition, but this turned out to be due to agglutination of the bacteria rather than any inhibition of bacterial enzyme activities [17]. The term antiblastic immunity quickly grew out of fashion and wasn’t used anymore, also not by Avery.

When Avery and Dochez studied patients with lobar pneumonia, they found a “specific soluble substance” not only in blood but also in urine [18]. Subsequently, Heidelberger and Avery demonstrated that this specific soluble substance is, in fact, the capsular polysaccharide of the pneumococcus [19,20], which is the basis for the currently still widely used point-of-care test for pneumococcal pneumonia [21].

When Avery and Heidelberger purified the capsular polysaccharide, they demonstrated that (rabbit) antibodies against the polysaccharide could protect mice against an otherwise lethal dose of live pneumococci [22]. They were unable, however, to induce the antibodies with the purified polysaccharide itself but they needed the intact, heat-killed bacterium to do so. In the discussion of their paper, they therefore state that “in the case of Pneumococcus it has been shown that the polysaccharides by themselves are not antigenic” [22]. Later studies by others, however, did show that polysaccharides by themselves are immunogenic, and polysaccharide-based vaccines for pneumococci, meningococci and other polysaccharides have been in use for decades [23]. The reasons why Avery and Heidelberger were unsuccessful are unclear, but it could have to do with dosage, route of administration, lack of an adjuvant or otherwise. It may have been the reason why Avery, with Walther Goebel, set out a series of experiments to improve the immunogenicity of polysaccharides. They found that the chemical coupling of bacterial sugars of pneumococcus to a specific protein greatly increases the immunogenicity [24,25].

The report of their experiments was split into two parts; part I focused on the coupling of the sugars [24] while the second reported the immune response in rabbits [25]. They discovered that not only was it possible to couple these polysaccharides to proteins [24] but these protein-sugar conjugates were highly immunogenic in their test subjects [25]. For over 50 years, the true impact of this finding was overlooked. The polysaccharide vaccines used during those decades were unable to induce a response in young children, and thus polysaccharide-based vaccines are ineffective for this age group. It took until the 1980s before the principle of Avery and Goebel was put into practice and the capsular polysaccharides of *Haemophilus influenzae* type b, *Neisseria meningitidis* and *Streptococcus pneumoniae* were coupled to proteins to create so-called conjugate vaccines. Conjugate vaccines are important for the prevention of pneumonia, meningitis and sepsis because pure polysaccharide-based vaccines do not activate T-cells, which makes them ineffective in the young and less effective in the elderly and immunocompromised. Conjugated vaccines, however, specifically activate T-cells, which leads to a strong and lasting response in all risk groups [26]. Currently, the best-known vaccine is the pneumococcal conjugate vaccine (PCV), which is administered in three doses under the age of one year old and can protect against up to 20 serotypes of pneumococcus [27]. In 2022, 164 out of the 194 WHO member states have implemented PCVs into their National Vaccination Program for children. Since their introduction, these vaccines have saved the lives of millions of children [28].

Legacy

In his career, Oswald Avery held many accolades and positions. Avery served as President of the American Association of Immunologists (1929-1930), the American Association of Pathologists and Bacteriologists (1934) and the Society of American Bacteriologists (1941-1942). He was elected to the National Academy of Sciences in 1933 and the Royal Society of London in 1944. He received honorary degrees from, among others, McGill University, New York University, the University of Chicago and Rutgers University. In 1932, he received the John Phillips Memorial Award from the American College of Physicians; in 1945, the Copley Medal from the Royal Society of London; in 1946, the Kober Medal from the Association of American Physicians; and the New York Academy of Medicine. Avery received the Lasker Award in Basic Medical Research in 1947.

## Conclusions

Oswald Avery’s contributions to science and medicine are immense. His discovery of DNA being the carrier of genetic information challenged the prevailing paradigm. Like other groundbreaking discoveries, his conclusions were at first both questioned and denied. His other, earlier discovery of the superior immunogenicity of polysaccharide antigens when coupled to protein carriers was not so much questioned or denied but ignored for 50 years. Oswald Avery was recognized with the Lasker Award. Since 1945, 86 Lasker laureates have received the Nobel Prize, but Oswald Avery didn’t. The archives of the Nobel Committee show that he was nominated 38 times. In an obituary of Oswald Avery, René J. Dubos expressed his hopes that at one point in time, the Nobel Academy would acknowledge in public this oversight. They could take the example of the Académie Française in the case of Molière: “Rien ne manquait a sa gloire, il manquait a la notre” (nothing was missing from his glory, it was missing from ours). Unfortunately, in 2024, that point in time hasn’t come yet for the Nobel Academy.
